# LPS Induces GM-CSF Production by Breast Cancer MDA-MB-231 Cells via Long-Chain Acyl-CoA Synthetase 1

**DOI:** 10.3390/molecules25204709

**Published:** 2020-10-14

**Authors:** Fatema Al-Rashed, Reeby Thomas, Areej Al-Roub, Fahd Al-Mulla, Rasheed Ahmad

**Affiliations:** 1 Immunology & Microbiology Department, Dasman Diabetes Institute, Kuwait City 15462, Kuwait; fatema.alrashed@dasmaninstitute.org (F.A.-R.); reeby.thomas@dasmaninstitute.org (R.T.); areej.abualroub@dasmaninstitute.org (A.A.-R.); 2 Genetics and Bioinformatics, Dasman Diabetes Institute, Kuwait City 15462, Kuwait; fahd.almulla@dasmaninstitute.org

**Keywords:** LPS, ACSL1, GM-CSF, MDA-MB-231, MAPK

## Abstract

Granulocyte–macrophage colony-stimulating factor (GM-CSF) is a monomeric glycoprotein that has been implicated in the tumor growth and progression of different types of cancer. GM-CSF is produced by various non-immune cells including MDA-MB-231 in response to various stimuli. However, the role of lipopolysaccharide (LPS) in the regulation of GM-CSF in MDA-MB-231 breast cancer cells so far remains unclear. Herein, we asked whether LPS could induce GM-CSF production in MDA-MB-231 cells, and if so, which signaling pathway was involved. MDA-MB-231 cells were treated with LPS or tumor necrosis factor alpha (TNF-α; positive control), and GM-CSF expression levels were determined by qRT-PCR, ELISA, and confocal microscopy. Phosphorylation of the mitogen-activated protein kinases (MAPKs) and nuclear factor-κB (NF-kB) signaling proteins were evaluated by flow cytometry. Our results show that LPS induces GM-CSF expression at both mRNA and protein levels in MDA-MBA-231 cells. Inhibition of acyl-CoA synthetase 1 (ACSL1) activity in the cells with triacsin C significantly reduces the secretion of GM-CSF. Furthermore, the inhibition of ACSL1 activity significantly blocks the LPS-mediated phosphorylation of p38 MAPK, MEK1/2, extracellular signal-regulated kinase (ERK)1/2, c-Jun NH2-terminal kinase (JNK), and nuclear factor-κB (NF-kB) in the cells. These findings provide the first evidence that LPS induces ACSL1-dependent GM-CSF gene expression in MDA-MB-231 breast cancer cells, which requires the activation of p38 MAPK, MEK1/2, ERK1/2, JNK, and NF-kB.

## 1. Introduction

Granulocyte-macrophage colony-stimulating factor (GM-CSF) is a monomeric glycoprotein/cytokine that is involved in the immune modulation and hematopoiesis [1]. GM-CSF is produced by activated monocytes, macrophages, T cells, B cells, fibroblasts, mast cells, vascular endothelial cells, and a wide variety of cancer cell types, including MDA-MB-231 [2,3] in response to various stimuli. The excessive production of GM-CSF is involved in chronic inflammatory disorders by maintaining the existence of target cells and promoting the renewal of macrophages [4]. In this regard, a pathogenic role of increased GM-CSF has been well established in autoimmune diseases that are associated with cellular immune responses such as multiple sclerosis (MS) and rheumatoid arthritis (RA) [5,6]. Many studies show that GM-CSF is involved in promoting tumor growth and progression. GM-CSF regulates cancer cell proliferation and migration in various types of cancer. Constitutive GM-CSF protein expression and secretion along with its association with tumor growth and poor prognosis have been noted in multiple cancer models [7,8,9]. Elevated GM-CSF in serum underlies obesity-associated breast cancer metastasis. Obesity increases the risk for the promotion and progression of various cancers. One important mechanism leading this association is the obesity-induced chronic low-grade inflammation. Overnutrition and high-fat and high-energy diets have been shown to facilitate the absorption of bacterial lipopolysaccharide (LPS) from intestinal bacteria in the stings of obesity [10,11]. It has been reported that increased inflammatory mediators contribute to the aggressive breast cancer phenotype in obesity, which increases the risk of mortality in breast cancer patients [12].

The inflammatory state of obesity enhanced GM-CSF production in obese cancer patients, and it led to promote tumor growth and progression. GM-CSF regulates cell proliferation and migration in various types of cancer, including breast cancer, and it is upregulated by various stimuli. It remains unclear how GM-CSF production is upregulated in obese breast cancer patients. Since LPS and GM-CSF are elevated in a state of obesity, we asked whether LPS could trigger GM-CSF production in MDA-MB-231 cells. In addressing our study’s hypothesis that LPS could activate the GM-CSF gene expression in MDA-MB-231 cells, herein we report that LPS induces GM-CSF expression in MDA-MB-231 cells, and it involves the activation of acyl-CoA synthetase 1 (ACSL1), p38 mitogen-activated protein kinase (MAPK), MEK1/2, extracellular signal-regulated kinase (ERK1/ERK2), c-Jun NH2-terminal kinase (JNK) and nuclear factor-κB (NF-kB).

## 2. Results

### 2.1. LPS Induces GM-CSF Gene Expression in Human MDA-MB-231 Cells

**Figure 1:**
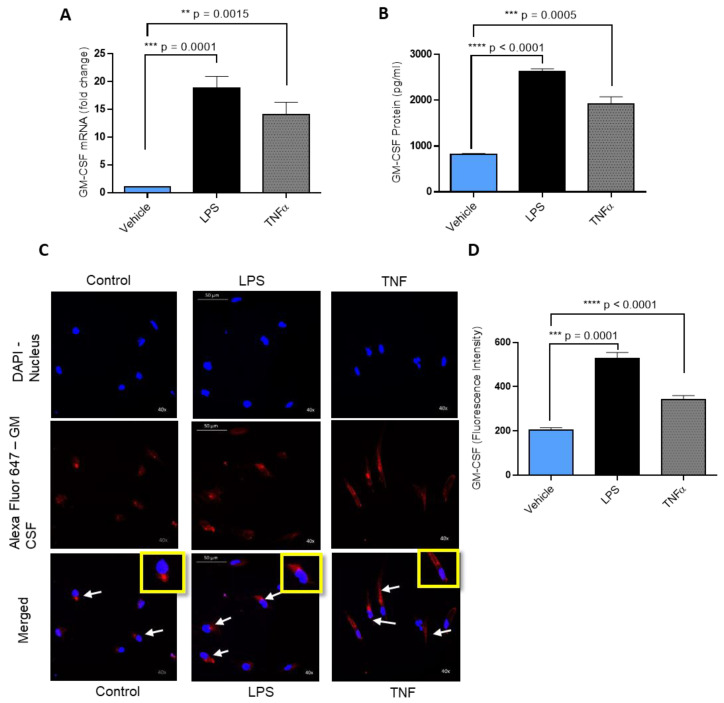
Effect of lipopolysaccharide (LPS) on granulocyte–macrophage colony-stimulating factor (GM-CSF) production in human MDA-MB-231 cells. MDA-MB-231 cells were cultured in six-well plates at a concentration of 1 × 10^6^ cells/well. Cells were treated with vehicle, LPS (10 ng/mL), and TNF-α (10 ng/mL; positive control) separately. After 24 h incubation, cells and supernatants were collected. (**A**) Total cellular RNA was isolated, and GM-CSF mRNA expression was determined by real-time RT-PCR. (**B**) Secreted GM-CSF protein in culture media was determined by ELISA. (**C**) MDA-MB-231 cells were treated with vehicle, LPS, or TNF-α for 24 h and then were stained with GM-CSF (red) and 4′,6-diamidino-2-phenylindole (DAPI) (blue). White arrows indicate typical stained cells. (**D**) GM-CSF fluorescence intensity was determined for 10 random images. The results obtained from three independent experiments are shown. All data are expressed as mean ± SEM (n ≥ 3). ** *p* < 0.01, *** *p* < 0.001, **** *p* < 0.0001 versus vehicle.

GM-CSF supports angiogenesis in primary breast tumor and further triggering cancer invasion and metastatic spread [3]. Elevated GM-CSF in serum underlies obesity-associated breast cancer metastasis. Since LPS and GM-CSF levels are higher in obesity settings, we wanted to investigate whether LPS induced GM-CSF production in MDA-MB-231 cells. This was accomplished by exposing MDA-MB-231 cells to vehicle or LPS or TNF-α (as a positive control) for 24 h. Our data show that LPS treatment of the MDA-MB-231 cells induced high GM-CSF mRNA expression level compared to the cells treated with vehicle (Figure 1A). We also measured the GM-CSF protein level in the supernatant of the MDA-MB-231 cells incubated with vehicle or LPS or TNF-α. Similar to mRNA, GM-CSF secreted protein levels were significantly higher in MDA-MB-231 cells supernatant when stimulated with LPS (Figure 1B). Confocal microscopy along with the fluorescence intensity of MDA-MB-231 cells also showed that there was a significant increase in the expression of GM-CSF in the cells treated with LPS or TNF-α (positive control) (Figure 1C,D).

### 2.2. LPS-Induced GM-CSF Production Is Downregulated by the Inhibition of ACSL1

**Figure 2:**
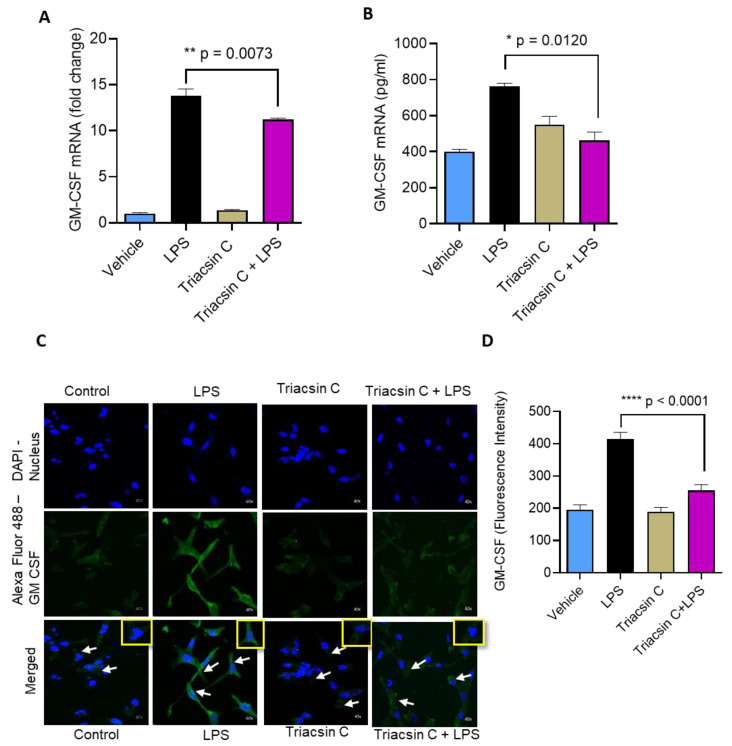
Effect of acyl-CoA synthetase 1 (ACSL1) inhibition on LPS mediated GM-CSF production in MDA-MB-231 cells. MDA-MB-231 cells were pretreated with a long chain acyl-CoA synthetase (ACSL1) inhibitor (triacsin C: (1 μM) or vehicle for 1 h and then incubated with LPS for 24 h. (**A**) Total cellular RNA was isolated, and GM-CSF mRNA expression was determined by real-time RT-PCR. (**B**) Secreted GM-CSF protein in culture media was determined by ELISA. (**C**) Representative figures for confocal microscopy. MDA-MB-231 cells were stained with GM-CSF (green) and DAPI (blue). White arrows indicate typical stained cells. White arrows indicate typical stained cells. (**D**) GM-CSF fluorescence intensity was determined for 10 random images. All data are expressed as mean ± SEM (n ≥ 3). * *p* < 0.05, ** *p* < 0.02, **** *p* < 0.001 versus vehicle.

GM-CSF production requires ACSL1 when MDA-MB-231 cells are stimulated with TNFα. To see whether the inhibition of ACSL1 activity would influence the expression of GM-CSF, MDA-MB-231 cells were treated with triacsin C prior to LPS treatment. ACSL1 inhibition with triacsin significantly suppressed the LPS-mediated production of GM-CSF by MDA-MB-231 cells (Figure 2A,B). We also confirmed these findings with confocal microscopy (Figure 2C). Confocal microscopy data revealed that the fluorescence intensity of GM-CSF staining content was reduced in MDA-MB-231 cells pretreated with triacsin C following exposure to LPS (Figure 2D).

### 2.3. ACSL1 Is Involved in LPS Activated MAPK and NF-kB Signaling Pathways

**Figure 3:**
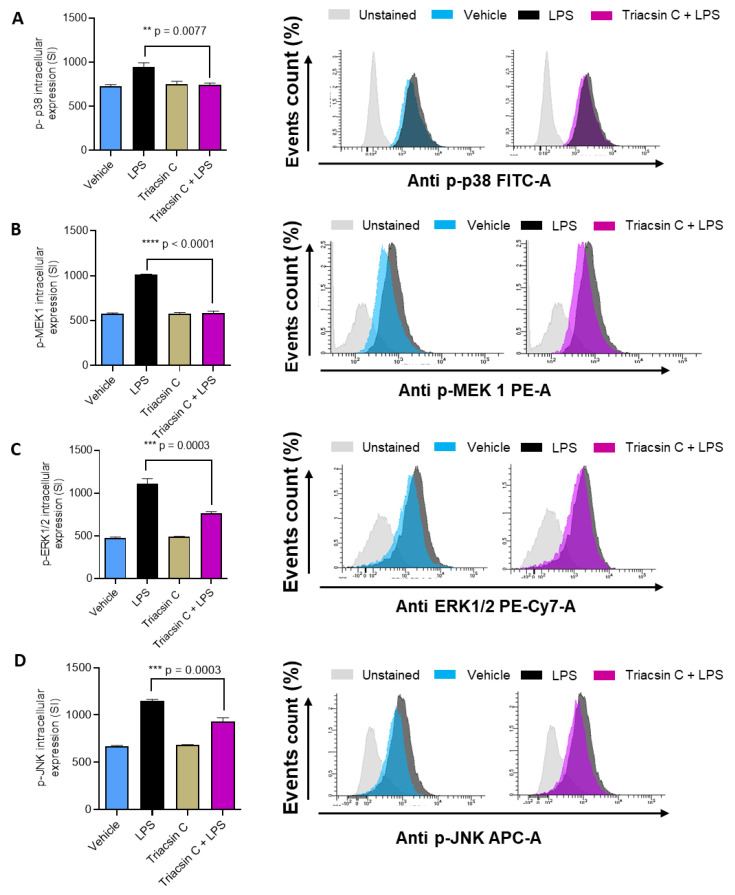
Mitogen-activated protein kinase (MAPK) signaling molecules activation in LPS-treated MDA-MBA-231 was inhibited by triacsin C. MDA-MBA-231 cells were pretreated with ACSL1 inhibitor (Triacsin C: (1 μM) or vehicle for 1 hr and then incubated with LPS for 15 min. There is no difference between vehicle and Triacsin C cells. Therefore, for the histogram, we used only vehicle-treated cells. (**A**–**D**) The phosphorylation of p38, extracellular signal-regulated kinase (ERK1/2) and c-Jun NH2-terminal kinase (JNK) was determined by flow cytometry presented in the form of staining intensity and representative histograms. The data are representative of three independent experiments. All data are expressed as mean ± SEM (n ≥ 3). ** *p* < 0.01, *** *p* < 0.001, **** *p* < 0.0001 versus LPS.

**Figure 4:**
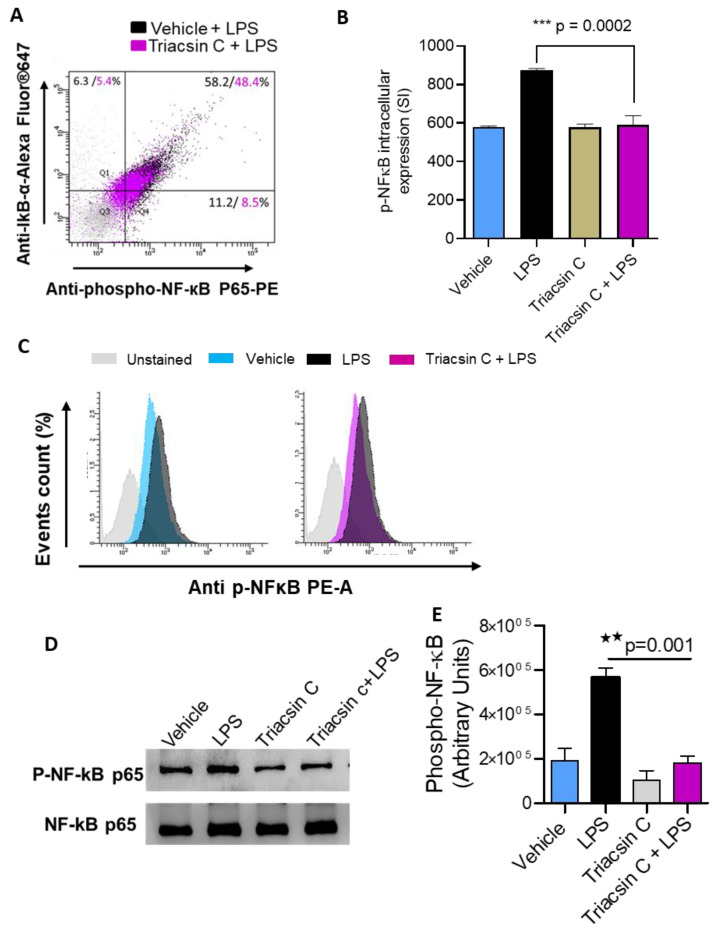
Nuclear factor-κB (NF-κB) activation in LPS-treated monocytes was inhibited by triacsin C. MDA-MB-231 cells were pretreated with ACSL1 inhibitor (Triacsin C: (1 μM) or vehicle for 1 h and then incubated with LPS for 15 min. NF-κB phosphorylation was determined by flow cytometry. (**A**) Representative flow cytometry dot plots of p-NF-κB fluorescence versus total inhibitor of kappa B protein alpha (IkBα) cells. (**B**) Staining intensity of flow cytometry analysis of NF-kB phosphorylation. (**C**) Representative histograms of one experiment. (**D**) MDA-MB-231 were pretreated with ACSL1 inhibitor (Triacsin C: (1 μM) or vehicle for 1 hr and then incubated with LPS for 15 min. Cell lysates were prepared as described in Materials and Methods. Samples were run on denaturing gels. Phosphorylated NF-κB is depicted in the upper panel, and total respective proteins are shown in the lower panel. (**E**) Phosphorylation intensity of NF-κB was quantified using Image Lab software (version 6.0.1, Bio-Rad, Hercules, CA, USA) and are presented in arbitrary units, which indicated that the Western blot protein data for phospho NF-KB and NF-kB clearly exhibited a similar treatment response to that seen that by flow cytometry. All data are expressed as mean ± SEM (n ≥ 3). ** *p* < 0.01, *** *p* < 0.001 versus LPS.

LPS activate a network of signaling pathways that influence the expression of several genes. It is known that LPS stimulates the activation of mitogen-activated protein kinases (MAPKs) (p38, MEK1/2, ERK1/2, JNK) and NF-kB signaling pathways [13,14]. In agreement with these reports, our flow cytometry data show that LPS treatment of MDA-MB-231 induces the phosphorylation of p38 MAPK, MEK1/2, ERK1/2, JNK (Figure 3A–D) and NF-kB; Figure 4A–D). Next, we explore whether ACSL1 is involved in the LPS-mediated phosphorylation of p38 MAPK, ERK1/2, JNK, and NF-kB. MDA-MB-231 cells were treated with LPS in the presence or absence of triacsin C and the phosphorylation of key signaling molecules was determined by flow cytometry. Our flow cytometry results showed that ACSL1 inhibition with triacsin C significantly attenuated the LPS-induced phosphorylation of p38 MAPK, ERK1/2, JNK (Figure 3A–D), and NF-kB (Figure 4A–C). Our Western blot protein data for phospho NF-KB and total NF-kB clearly exhibited a similar treatment response (Figure 4D,E) to that seen that by flow cytometry. Overall, our results are signifying that p38 MAPK, ERK1/2, and NF-kB molecules were downstream of LPS/ACSL1 signaling.

**Figure 5:**
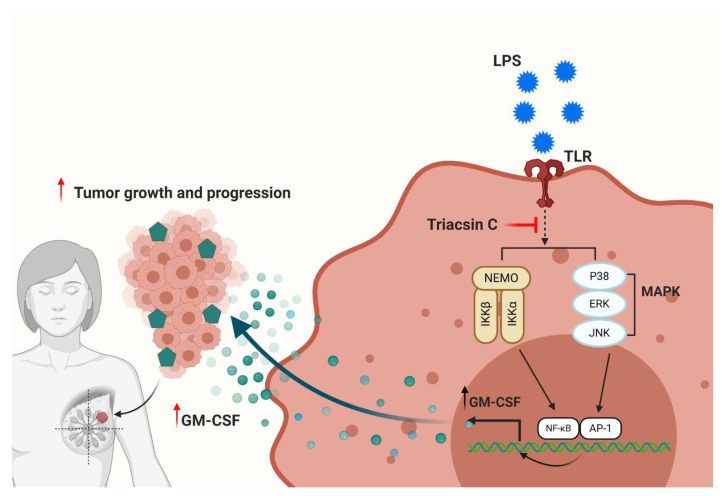
A novel role of ACSL1 in LPS induced GM-CSF production in MDA-MB-231 breast cancer cells. LPS: Lipopolysaccharide; ACSL1: long-chain acyl-CoA synthetase 1; p38 MAPK: p38 mitogen-activated protein kinase ERK: extracellular signal-regulated kinase; NF-κB: nuclear factor kappa-light-chain-enhancer of activated B cells; GM-CSF: granulocyte-macrophage colony-stimulating factor.

The thematic illustration presented below summarizes the aforementioned findings of this study (Figure 5).

## 3. Discussion

In this study, we report for the first time that LPS induced GM-CSF production by MDA-MB-231 cancer cells. LPS regulates the production of IL-6, IL-8, and GM-CSF in monocytic cells [15], which supported our findings. In addition, a growing body of evidence suggests that GM-CSF is produced and secreted by a wide variety of non-immune cell types, including fibroblasts, keratinocytes, endothelial cells, and MDA-MB-231 cells in response to appropriate stimuli [3,16]. LPS activates inflammatory responses in monocytes/macrophages via ACSL1 [17]. Our data showed that the inhibition of ACSL1 significantly blocked the LPS-induced production of GM-CSF, which is supported by the study that ACSL1-deficient mouse macrophages display reduced inflammatory responses after long-term stimulation with LPS [18].

Regarding molecular mechanisms underlying the LPS-induced production of GM-CSF, It is known that LPS induces the activation and phosphorylation of MAP kinase signaling pathways (p38, ERK1/2, JNK) [13,14] that regulate the expression of several genes. In agreement with previous reports, our flow cytometry data show that LPS treatment of MDA-MB-231 induces the phosphorylation of p38 MAPK, ERK1/2, and JNK. It has been reported that ACSL1 function in TNF-α induced the activation of MAPK and NF-κB signaling proteins. It is well documented that TNF-α stimulates MAPK and NF-κB signaling pathways involved in the regulation of several inflammatory cytokines that contribute to the pathogenesis of different inflammatory conditions including state of obesity [19,20], suggesting the role of ACSL1 and its association with MAPKs/NF-kB signaling pathways in the development of chronic low-grade inflammation. LPS also plays a key role in the activation of MAPKs and NF-kB signaling pathways and interestingly, our results for the first time show that the inhibition of ACSL1 reduced the LPS-induced activation of MAPK and NF-kB signaling proteins in a similar pattern, as we previously demonstrated that the disruption of the activity of ACSL1 in MDA-MB-231 suppressed the phosphorylation of p38 MAPK, ERK1/2, and JNK ([3]). Thus, ACSL1 appears to play a central role in MDA-MB-231 for the LPS-mediated activation of MAPK signaling pathways in parallel to the production of GM-CSF. It is well documented that LPS stimulates MAPK signaling pathways involved in the regulation of several inflammatory cytokines that contribute to the pathogenesis of different inflammatory conditions. Meja et al. reported that LPS-induced GM-CSF secretion by human monocytic cells was partially blocked by p38 MAPK and ERK1/2 [21].

The transcription factor NF-κB induces the expression of various pro-inflammatory genes, including those encoding cytokines and chemokines. LPS induced NF-kB activation and cytokine (IL-6 and IL-8) production in human myeloid and non-myeloid cells [22]. The dysregulation of NF-κB activation contributes to the pathogenic processes of various inflammatory diseases [23]. Our results showed that the inhibition of ACSL1 activity reduces the phosphorylation of NF-kB in response to LPS, indicating a very interesting role of ACSL in LPS-mediated activation of major transcription factor NF-kB, which was supported by the findings indicating that ACSL inhibition attenuates NF-κB activity resulting from TNF-α stimulation [3]. These results indicate that ACSL1 acts upstream of the NF-κB pathway in a similar fashion as noted in case of GM-CSF production by TNF-α [3]. We speculate that LPS interacts with MDA-MB-231 cells and activates a complex process, which includes the participation of several molecules including MAPK signaling molecules (p38, ERK1/2, JNK, C-Jun) interacting with each other along with having extensive cross-talk to other inflammatory pathways (including NF-kB) in the orchestration of inflammatory responses that regulate GM-CSF.

In summary, our findings show that LPS induces the production of GM-CSF by MDA-MB-231 breast cancer cells. Furthermore, our study demonstrates a distinct role of ACSL1 in the regulation of LPS-mediated GM-CSF production in MDA-MB-231 cells. Interestingly, ACSL1 acts upstream of p38, ERK1/2, JNK, and NF-kB signaling molecules.

## 4. Material and Methods

### 4.1. Cell Culture

Human MDA-MB-231 cells were purchased from the American Type Culture Collection (ATCC), grown in Dulbecco’s Modified Eagle Medium (DMEM) culture medium (Gibco, Life Technologies, Grand Island, NE, USA) supplemented with 10% fetal bovine serum (Gibco, Life Technologies, Grand Island, NE, USA), 2 mM glutamine (Gibco, Invitrogen, Grand Island, NE, USA), 1 mM sodium pyruvate, 10 mM N-2-hydroxyethylpiperazine-N-ethanesulfonic acid (HEPES), 100 ug/mL normocin, 50 U/mL penicillin, and 50 μg/mL streptomycin (P/S; (Gibco, Invitrogen, Grand Island, NE, USA), and incubated at 37 °C (with humidity) in 5% CO_2_.

### 4.2. Cell Stimulation

MDA-MB-231 cells were plated in 12-well plates (Costar, Corning Incorporated, New York, NY, USA) at 1 × 10^6^ cells/well concentration unless indicated otherwise. Cells were treated with vehicle or TNF-α (2 ng/mL; 210-TA, R&D Systems, Minneapolis, MN, USA) or lipopolysaccharide (LPS) (10ng/mL; L4391, Sigma Aldrich, Merck KGaA, Darmstadt, Germany) for 24 h at 37 °C. After incubation, cells were harvested for RNA isolation, and conditioned media were collected for measuring secreted GM-CSF. Then, cells were stimulated with TNF-α (2 ng/mL; 210-TA, R&D Systems, Minneapolis, MN, USA) or lipopolysaccharide (LPS) (10 ng/mL; L4391, Sigma Aldrich, Merck KGaA, Darmstadt, Germany) overnight at 37 °C unless otherwise specified.

### 4.3. Real-Time Quantitative RT-PCR

Total RNA was extracted from MDA-MB-231 cells using a RNeasy Mini Kit (Qiagen, Valencia CA, USA) per the manufacturer’s instructions [24]. The cDNA was synthesized using 1 μg of total RNA using a high-capacity cDNA reverse transcription kit (Applied Biosystems, Foster city, CA, USA). Real-time PCR was performed on a 7500 Fast Real-Time PCR System (Applied Biosystems, Foster City, CA, USA) using TaqMan^®^ Gene Expression Master Mix (Applied Biosystems). Each reaction contained 500 ng of cDNA that was amplified with Inventoried TaqMan Gene Expression Assay products (CSF2: Hs00929873; ACSL1: Hs00960561; GAPDH: Hs03929097_g1). The threshold cycle (Ct) values were normalized to the housekeeping gene GAPDH, and the amounts of target mRNA relative to control were calculated with the ΔΔCt method [25,26]. Relative mRNA expression was expressed as fold expression over average of control gene expression. The expression level in control treatment was assumed to be 1. Values are presented as mean ± SEM. Results were analyzed statistically; *p* < 0.05 was considered significant [27,28].

### 4.4. Intracellular Staining and Flow Cytometry

Human MDA-MB-231 cells were pretreated with ACSL1 inhibitor (Triacsin C: (1 μM) or vehicle for 1 h and then incubated with LPS for 15 min. The samples were fixed and permeabilized with a BD Cytofix/Cytoperm™ fixation/permeabilization kit (Cat no. 554714, BD Biosciences, San Jose, CA, USA) according to the manufacturer’s protocol at 4 °C for 15 min. Then, the cells were washed 2 times in cold phosphate-buffered saline (PBS), and the permeabilized cells were incubated with 5 μL of phosphorylated antibodies against p38 MAPK Alexa Fluor^®^ 488 (Cat no. 612594), MEK1 PE (Cat no. 560099), ERK1/2 PE-Cy7 (Cat no. 560116), JNK Alexa Fluor^®^ 647 (Cat no. 562481), IκBα Alexa Fluor^®^ 647 (Cat no. 560817), and NFκB PE (Cat no. 558423) antibodies for 20 min in 4 °C. All antibodies were purchased from Biosciences, San Jose, CA, USA. The cells were washed twice in FACS buffer (PBS containing 5% FBS and 2mM EDTA) and resuspended in FACS buffer and analyzed using BD FACSDiva Software (V.8.0.2, BD Biosciences San Jose, CA, USA).

### 4.5. GM-CSF Determination

Secreted GM-CSF protein in supernatants of MDA-MB-231 cells stimulated with TNF-α was quantified using sandwich ELISA following the manufacturer′s instructions (DY215-05, R&D systems, Minneapolis, MN, USA).

### 4.6. Immunocytofluorescence

MDA-MB-231 cells (10^6^/mL) were seeded on coverslips and cultured in 6-well plates at 37 °C. After incubation, the slides were fixed in 4% formaldehyde and washed three times in cold PBS. Then, cells were permeabilized using in 0.1% Triton X-100, followed by three washes in cold PBS. The cells were blocked in 1% bovine serum albumin for 1 hr. The slides were incubated overnight at room temperature with a primary antibody of rabbit polyclonal anti GM-CSF antibody (GTX51383 Genetex, Irvine, CA, USA) in 1:200 dilution. Then, the cells were washed in PBS containing 0.05% Tween three times and again incubated with the secondary antibody conjugated with Alexa Fluor 647 or Alexa Fluor 488 (abcam^®^ ab150079, abcam^®^ ab150077) 1:200 dilution for 1 h. After washing the slide several times in PBS, the cells were counterstained and mounted with a coverslip using mountant containing DAPI (Vectashield, Vectorlab, H1500).

The confocal images of the MDA-MB-231 cells were collected on an inverted Zeiss LSM710 AxioObsever microscope (Carl Zeiss, Gottingen, Germany) using Plan-Apochromat 40X/1.40 oil DIC M27 objective lens. Excitation was via a 647 nm HeNe solid-state laser and the 405 nm line of an argon ion laser. After laser excitation of the samples, optimized emission detection bandwidths were configured by Zeiss Zen 2010 control software; subsequently, the confocal images were captured, and fluorescence was measured using Zeiss Zen 2012 software.

### 4.7. Western Blotting

MDA-MB-231 cells were treated with LPS and incubated for 30 min with lysis buffer (10X Lysis Buffer, Cell Signaling, Danvers, MA, USA). The protein lysates were prepared and resolved by 12% SDS-PAGE, as described earlier [26]. Cellular proteins were transferred to an Immuno-Blot polyvinylidene difluoride (PVDF) membrane (Bio-Rad Laboratories, Hercules, CA, USA) by electroblotting. Then, the membranes were blocked with 5% non-fat milk in PBS for 1 h, which was followed by incubation with primary antibodies against p-NF-κB (cat# 3033) and NF-κB (cat# 3034) in 1:1000 dilution at 4 °C overnight. All primary antibodies were purchased from Cell Signaling (Cell Signaling Technology Inc., Danvers, MA, USA). Then, the blots were washed three times with Tris-buffered saline with tween (TBS-T) and incubated for 2 h with HRP-conjugated secondary antibody (Promega, Madison, WI, USA). Immunoreactive bands were developed using an Amersham ECL Plus Western Blotting Detection System (GE Healthcare, Chicago, IL, USA) and visualized by Molecular Imager^®^ VersaDocTM MP Imaging Systems (Bio-Rad Laboratories, Hercules, CA, USA).

## 5. Statistical Analysis

GraphPad Prism software (La Jolla, CA, USA) was used for statistical analysis. Data were presented as mean ± standard error of the mean (SEM). For comparison between means of the groups, an unpaired Student *t*-test and one-way ANOVA were used followed by Tukey′s test. *p* value < 0.05 was considered significant. Ns: no significance, * *p* < 0.05, ** *p* < 0.01, *** *p* < 0.001 and **** *p* < 0.0001).
